# The Association of CSF sTREM2 With Cognitive Decline and Its Dynamic Change in Parkinson's Disease: Analysis of the PPMI Cohort

**DOI:** 10.3389/fnagi.2022.892493

**Published:** 2022-06-16

**Authors:** Qixiong Qin, Hengming Wan, Danlei Wang, Jingyi Li, Yi Qu, Jingwei Zhao, Jiangting Li, Zheng Xue

**Affiliations:** ^1^Department of Neurology, Tongji Hospital, Tongji Medical College, Huazhong University of Science and Technology, Wuhan, China; ^2^Department of General Family Medicine, Liuzhou Worker's Hospital, The Fourth Affiliated Hospital of Guangxi Medical University, Liuzhou, China

**Keywords:** Parkinson's disease, triggering receptor expressed on myeloid cells 2 (TREM2), cognitive decline, Parkinson's Progression Markers Initiative (PPMI), cerebrospinal fluid

## Abstract

**Background:**

Soluble fragment of triggering receptor expressed on myeloid cells 2 (sTREM2) in cerebrospinal fluid (CSF) is a biomarker of microglial activation and increased in several neurodegenerative diseases. However, the role of sTREM2 in Parkinson's diseases (PDs) remains unclear. This study aims to investigate whether CSF sTREM2 is changed during the pathology of PD and its association with cognitive decline.

**Methods:**

We recruited 219 *de novo* patients with PD and 100 healthy controls from Parkinson's Progression Markers Initiative (PPMI). Cross-sectional and longitudinal associations between cognition and CSF sTREM2 were evaluated using multivariable-adjusted models. To assess the changes in CSF sTREM2 during the pathology of PD, patients were classified through the A/T classification framework with addition of α-synuclein (α-syn), which we implemented based on the CSF amyloid β-peptide _1−42_ (A) and phosphorylated tau (T) and α-syn (S).

**Results:**

The CSF sTREM2 did not differ between healthy controls and patients with PD or between PD clinical subgroups (*p* > 0.05). However, higher baseline CSF sTREM2 predicted greater global cognitive decline in patients with PD (β = −0.585, *p* = 0.039). Moreover, after a mean follow-up of 5.51 ± 1.31 years, baseline CSF sTREM2 that elevated in the middle tertile (HR = 2.426, 95% CI: 1.023–5.754, *p* = 0.044) and highest tertile (HR = 2.833, 95% CI: 1.226–6.547, *p* = 0.015) were associated with a future high risk of cognitive decline. Additionally, CSF sTREM2 decreased in abnormal Aβ pathology (A+) and α-syn pathology (S+) but normal tau pathology, while increased in abnormal phosphorylated tau (T+) (*p* < 0.05).

**Conclusion:**

CSF sTREM2 may be a promising predictor for the cognitive decline in PD rather than a diagnostic biomarker. The dynamic change in CSF sTREM2 in PD may help to the monitor of neuronal injury and microglial activity.

## Introduction

Parkinson's disease (PD) is a common neurodegenerative disorder characterized by abnormal aggregation of α-synuclein (α-syn), progressive loss of dopaminergic neurons, and neuroinflammation (GBD, 2016). Motor symptoms and non-motor symptoms are two common clinical manifestations of PD, and cognitive decline is one of the non-motor symptoms that seriously affects the prognosis and quality of life (Bloem et al., 2021). It is reported that about 25% of patients with PD meet the criteria for mild cognitive impairment (MCI) at the time of diagnosis and up to 80% of patients eventually suffer from dementia (Muslimovic et al., 2005; Hely et al., 2008; Aarsland et al., 2009). Triggering receptor expressed on myeloid cells 2 (TREM2), an innate immune receptor expressed on microglia, is recently shown to be a specific marker of microglial activation (Deczkowska et al., 2020). In addition, neuroinflammation caused by overactivation of microglia is thought to be an important cause of PD and is associated with cognitive decline (De Virgilio et al., 2016; Zhang et al., 2021).

Previous studies had confirmed that TREM2 mutations increased the risk of Alzheimer's disease (AD) (Carmona et al., 2018), whereas the association of TREM2 mutations with PD remains controversial (Benitez and Cruchaga, 2013; Rayaprolu et al., 2013; Mengel et al., 2016). Nevertheless, the recent studies have verified that TREM2 is involved in the pathological process of PD animal models by regulating microglia activation, neuroinflammation, and α-syn clearance (Ren et al., 2018; Zhang et al., 2018; Guo et al., 2019). Cumulative studies have showed that soluble TREM2 (sTREM2) cleaved and released in cerebrospinal fluid (CSF) by microglia was elevated in elderly and patients with PD and was associated with cognitive decline (Suárez-Calvet et al., 2016; Ewers et al., 2019). Recently, CSF sTREM2 was found to be increased in patients with PD and was positively associated with CSF total α-syn (Peng et al., 2020; Mo et al., 2021). However, there were also studies showed that CSF sTREM2 was not increased in patients with PD (Wilson et al., 2020; Bartl et al., 2021). Therefore, the association of CSF sTREM2 with cognitive decline in PD remains controversial.

Due to longitudinal data were deficient in previous studies, the prediction of CSF sTREM2 on cognitive decline and its dynamic changes in PD are unclear. Therefore, by the means of Parkinson's Progression Markers Initiative (PPMI) database, this study aims to investigate the baseline and longitudinal changes of CSF sTREM2 in patients with PD and to assess its association with cognitive decline. Identifying the dynamic change in CSF sTREM2 and its relationship with cognitive decline in PD is critical for clinical management and the measurement of therapeutic efficacy in clinical trials.

## Methods

### Study Participants

Study participants were recruited from PPMI database, and the data analyzed included baseline and longitudinal data were collected from the PPMI database as of 28 July 2021 (https://www.ppmi-info.org/). PPMI is a multicenter, longitudinal, and observational cohort that aims to identify biological, clinical, and neuroimaging biomarkers of PD progression. This study was approved by the Institutional Review Board of all participating sites. In addition, written informed consent has been obtained from all participants. The study is registered in clinicaltrials.gov as NCT01141023.

The inclusion criteria for patients with PD were as follows: 1) an idiopathic PD diagnosis within 2 years, 2) age ≥ 30 years, 3) no dopaminergic treatment and no signs of dementia (Montreal Cognitive Assessment (MoCA) total score ≥ 22) at baseline, and 4) dopamine transporter deficit on SPECT. Patients were excluded if they: 1) were diagnosis with parkinsonian syndrome, hereditary PD, multiple system atrophy, progressive supranuclear palsy, or corticobasal ganglionic degeneration; 2) combined with encephalitis, epilepsy, traumatic brain injury, cancer, or severe psychiatric. The inclusion criteria for healthy controls were as follows: 1) age, gender, and education level matched with patients with PD, 2) no neurological disease, and 3) cognition normal, i.e., MoCA total score ≥ 26. All enrolled participants had an available baseline CSF sTREM2 and had at least one additional monitoring CSF sTREM2 during the follow-up.

### Measurements of CSF sTREM2

The levels of CSF sTREM2 were determined by electrochemiluminescence immunoassays (ECLIAs) on the fully automated cobas e411 with eight Roche Elecsys immunoassays. CSF Aβ_1−42_, P-tau, and T-tau were measured by ECLIA on a fully automated cobas e601 analyzer (Elecsys, Roche diagnostic). CSF alpha-synuclein (α-syn) was analyzed using an enzyme-linked immunosorbent assay (ELISA) available commercially from Covance (cat # SIG-38974-kit). *Apolipoprotein E* (*APOE*) genotypes were determined using allele-specific oligonucleotide probes labeled with fluorogenic reporter (TaqMan method). In addition, subjects were divided into *APOE* ε*4* carrier (*APOE* ε*4* +) and non-*APOE* ε*4* carrier (*APOE* ε*4*-) according to their *APOE* genotypes: the presence or absence of one or more ε*4* alleles (Kang et al., 2016). Detailed study protocols can be found at https://www.ppmi-info.org/.

### A/T/N Classification Framework

The A/T/N classification framework includes three biomarker groups: “A” refers to aggregated Aβ, “T” were aggregated tau, and “N” to neurodegeneration (Jack et al., 2018). Each biomarker group is dichotomized as negative (–) or positive (+) based on whether its biomarkers are normal or abnormal. Since PD and AD shared a certain degree of neuropathological overlap, we applied the cutoff point for amyloid positivity established in AD to distinguish “A+” and “A–.” To mitigate the differences in pre-analytical factors between PPMI and AD cohorts that influence CSF Aβ_1−42_ levels (Stewart et al., 2019), we applied the transformation formula to convert Elecsys values to AlzBio3 equivalents [x = (CSF Aβ_1−42_ + 251.55)/3.74] and used the established cutoff point <250 pg/ml (Shaw et al., 2018) of AlzBio3 equivalent values to designate “A+” or “A–,” which has been proposed in the recent studies analyzing of PPMI database (Irwin et al., 2020; Ma et al., 2021). We defined “T+” to individuals with P-tau181 > 21.8 pg/ml and “N+” to individuals with T-tau > 245 pg/ml (Ewers et al., 2019; Weinshel et al., 2022). Owing to T-tau reflecting neuronal injury (N) was highly correlated with the P-tau (*r* = 0.962, *p* < 0.001) (Buerger et al., 2006; Irwin et al., 2017) and only 3.13% (10/319) of all included PPMI participants had a discrepant P-tau and T-tau biomarker group, we did not take T-tau as the grouping reference to avoid statistical deviation caused by excessive grouping. Finally, participants were divided into four groups: A-T- (normal Aβ_1−42_ and P-tau), A+T- (low Aβ_1−42_, normal P-tau), A+T+ (low Aβ_1−42_, high P-tau), and A-T+ (normal Aβ_1−42_, high P-tau) according to A/T classification framework.

Evidence of cross-talk between CSF biomarkers (α-syn, P-tau, and T-tau) (Irwin et al., 2013; Lu et al., 2020) suggested that addition of PD biomarkers (α-syn) to A/T/N framework may help to improve monitoring CSF sTREM2 dynamics and cognitive decline in patients with PD. Since the cutoff value of CSF α-syn in PD has not been established, participants were dichotomized using the diagnostic cutoff value of CSF α-syn (≤ 107.7 pg/ml, Supplementary Figure S1) in PD in this study. We defined “S+” to individuals with α-syn ≤ 107.7 pg/ml, “S-” to individuals with α-syn >107.7 pg/ml. Finally, participants were divided into eight groups according to A/T classification framework with addition of CSF α-syn: A-T-S- (normal Aβ_1−42_, P-tau and α-syn), A-T-S+ (normal Aβ_1−42_ and P-tau, low α-syn), A+T-S- (low Aβ_1−42_, normal P-tau, and α-syn), A+T-S+ (low Aβ_1−42_, normal α-syn, high P-tau), A+T+S- (low Aβ_1−42_ and α-syn, normal P-tau), A+T+S+ (low Aβ_1−42_ and α-syn, high P-tau), A-T+S- (normal Aβ_1−42_ and α-syn, high P-tau), and A-T+S+ (normal Aβ_1−42_, high P-tau and low α-syn, *n* = 0).

### Clinical Assessment

The severity of motor symptoms was assessed by Unified Parkinson's Disease Rating Scale part III (UPDRS III) and Hoehn and Yahr (H&Y) stage. Cognitive assessment included global cognitive condition (Montreal Cognitive Assessment, MoCA total score), processing speed/attention (Symbol Digit Modality Test, SDMT), visuospatial abilities (Benton Judgment of Line Orientation, JoLO), executive function/working memory (Letter Number Sequencing, LNS), language (Semantic Fluency Test), and episodic memory (Hopkins Verbal Learning Test, HVLT) with total recall, delayed recall, retention, and recognition discrimination). The classification of cognitive status referred to the previous studies (Dalrymple-Alford et al., 2010; Litvan et al., 2012): normal cognition (NC, MoCA total score > 25), mild cognitive impairment (MCI, 22 ≤ MoCA total score ≤ 25), and dementia (MoCA total score <22). CSF biomarker information and cognitive assessment of participants during follow-up are shown in Supplementary Table S1.

### Statistical Analyses

Statistical analyses were performed by R 4.1.1 and IBM SPSS Statistics 26.0. Statistical significance was defined as two-tailed *p* < 0.05. Since CSF sTREM2, Aβ_1−42_, P-tau, T-tau, and α-syn were not normally distributed in Kolmogorov–Smirnov test, they were log10-transformed to obtain a normal distribution (Supplementary Figure S2). Log10-transformed values were used for all statistical of CSF sTREM2, Aβ_1−42_, P-tau, T-tau, and α-syn. To eliminate the effect of extreme values, outliers defined at baseline as three standard deviations above or below were excluded. Differences in characteristics between two groups were assessed by the Student's *t*-tests, Wilcoxon rank-sum tests, or χ^2^-test as appropriate. ROC analyses were performed to identify the optimal cutoff value for CSF α-syn in discriminating patients with PD from healthy controls. The associations of CSF sTREM2 with demographic characteristics were performed using the Spearman's rank correlation. Differences in CSF sTREM2 among groups were evaluated by one-way ANCOVA followed by Bonferroni postmortem analysis. Multiple linear regression model was used to assess the relationship of baseline CSF sTREM2 with CSF biomarkers and cognitive characteristics. Associations of baseline and longitudinal CSF sTREM2 with change rate of cognitive characteristics and CSF biomarkers were assessed by calculating normalized regression coefficients (β) in multiple linear regression models between slopes from linear mixed-effects (LME) models of CSF sTREM2 and cognitive characteristics and CSF core biomarkers. Slopes were calculated by a separate LME model. To assess the ability of CSF sTREM2 in prediction of cognitive decline, CSF sTREM2 was categorized into three groups (lowest tertile, middle tertile, and highest tertile) based on their distribution, which is referred to the method used in the previous studies (Ma et al., 2021; Lee et al., 2022). The cumulative incidence of cognitive progression among groups was compared using Kaplan–Meier curves. In addition Cox proportional-hazards regression models were used to analyze the correlation of CSF sTREM2 and cognitive progression. The models used in this study were adjusted for age, gender, education level, and *APOE* ε*4* carrier status.

## Results

### Participant Characteristics

**Figure 1:**
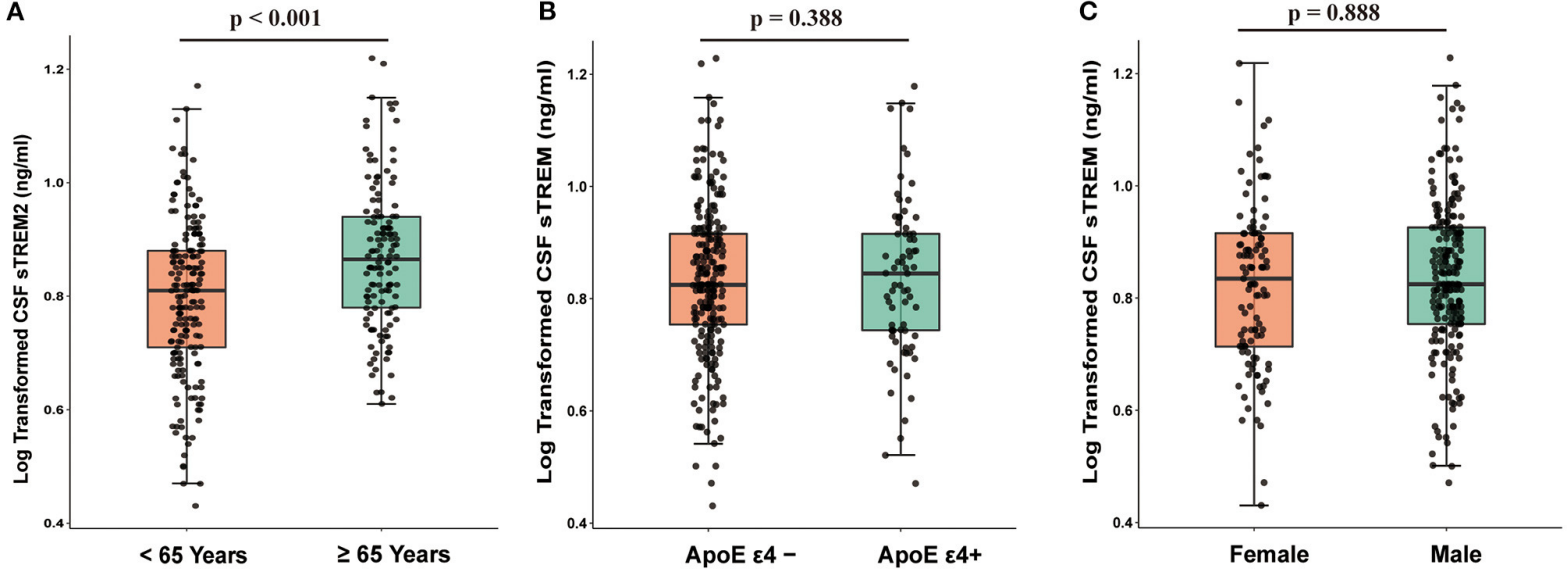
CSF sTREM2 was associated with age (*p* < 0.001; **(A)** while was independent of *APOE* ε*4* carrier status (*p* = 0.388; **(B)** and gender (*p* = 0.888; **(C)**. *p*-values were assessed by Student's *t*-test between two groups. CSF, cerebrospinal fluid; sTREM2, soluble triggering receptors expressed on myeloid cells 2.

The baseline characteristics of the participants are described in Table 1. In total, 219 patients with PD and 100 healthy controls (HC) were included. Compared to healthy controls, patients with PD had worse cognition and motor function and had lower CSF α-syn, T-tau, and P-tau (*p* < 0.05). CSF sTREM2 was significantly higher in elderly than middle-age in the whole cohort (n = 319; *t* = 4.106, *p* < 0.001, Figure 1A). The Spearman's rank correlation showed that CSF sTREM2 was positively correlated with age (*r* = 0.308, *p* < 0.001, Supplementary Figure S3). There was no difference in CSF sTREM2 between *APOE* ε*4* carrier status and gender (Figures 1B,C).

**Table 1:** Characteristics and CSF biomarkers of participants at baseline.

**Characteristics**	**HC (*n* = 100)**	**PD (*n* = 219)**	***p*-value**
Age (years)	62.02 ± 10.51	61.15 ± 9.82	0.476
Gender (F/M)	34/66	75/144	0.966
Education (years)	16.10 ± 2.61	16.19 ± 2.59	0.764
*APOE ε4* carrier status	27/94	46/203	0.259
Aβ_1−42_ (pg/ml)	991.36 ± 524.61	893.44 ± 400.17	0.055
T-tau (pg/ml)	192.08 ± 78.52	167.45 ± 56.68	0.004
P-tau (pg/ml)	17.53 ± 8.66	14.76 ± 5.16	0.005
α-synuclein (pg/ml)	126.49 ± 53.82	103.25 ± 47.76	<0.001
sTREM2 (ng/ml)	7.23 ± 2.42	6.97 ± 2.27	0.355
MoCA	28.11 ± 1.07	27.08 ± 2.15	<0.001
JoLO	13.13 ± 2.05	13.14 ± 1.86	0.951
LNS	10.78 ± 2.56	10.68 ± 2.56	0.732
SDMT	45.58 ± 10.81	42.09 ± 9.03	0.005
Semantic Fluency Test	51.57 ± 10.35	51.14 ± 9.78	0.719
HVLT Total Recall	49.37 ± 10.15	46.07 ± 10.38	0.008
HVLT Delayed Recall	48.04 ± 12.12	45.40 ± 10.84	0.052
HVLT Retention	48.28 ± 12.47	47.56 ± 11.46	0.611
HVLT RD	47.61 ± 12.01	45.40 ± 11.40	0.114
UPDRS-III	1.40 ± 2.27	20.77 ± 8.75	<0.001
H&Y	0.02 ± 0.14	1.59 ± 0.51	<0.001

*APOE, apolipoprotein E; Aβ_1−42_, amyloid_1−42_; T-tau, Total tau; P-tau, phosphorylated tau; α-syn, α-synuclein; sTREM2, soluble triggering receptors expressed on myeloid cells 2; MoCA, Montreal Cognitive Assessment; JoLO, Benton Judgment of Line Orientation Score; LNS, Letter Number Sequencing; SDMT, Symbol Digit Modality Test; HVLT, Hopkins Verbal Learning Test; HVLT RD, Recognition Discrimination; UPDRS-III, Unified Parkinson's Disease Rating Scale part III; H&Y, Hoehn and Yahr*.

### Baseline Associations of CSF sTREM2 With Cognitive Functions and CSF Biomarkers

**Figure 2:**
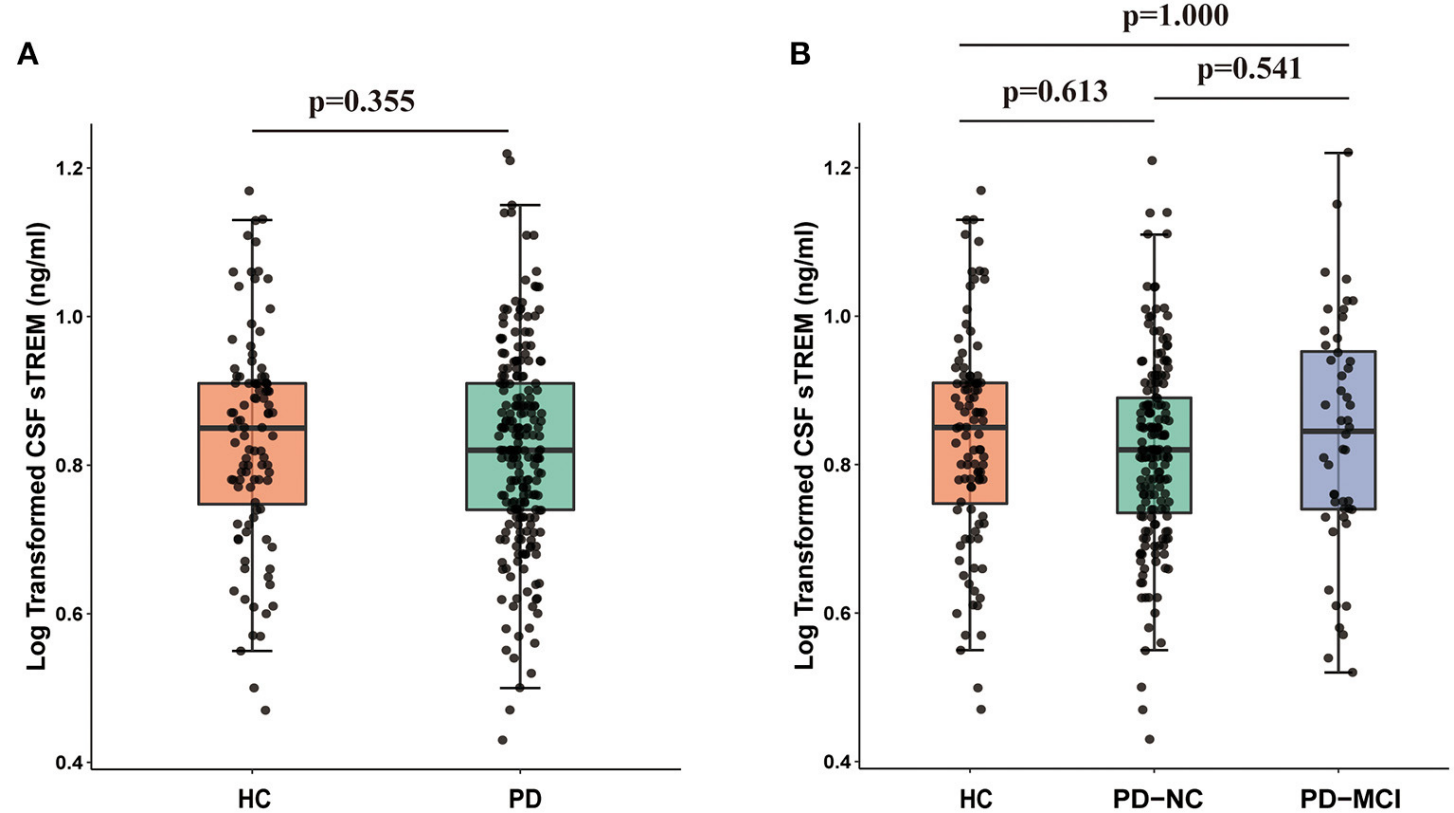
CSF sTREM2 was not different between HC group and PD group (*p* = 0.355; **(A)** or between HC group, PD-NC, and PD-MCI (*p* > 0.05; **(B)**. *p*-values were assessed by Student's *t*-test between two groups and one-way ANCOVA with Bonferroni corrected *post-hoc* pairwise comparisons among three groups. CSF, cerebrospinal fluid; sTREM2, soluble triggering receptors expressed on myeloid cells 2; HCs, healthy controls; PD, Parkinson's disease; NC, normal cognition; MCI, mild cognitive impairment.

There was no difference in CSF sTREM2 between PD group (6.98 ± 2.27 ng/ml) and HC group (7.23 ± 2.42 ng/ml) at baseline (Figure 2A). In addition, no difference was observed in CSF sTREM2 among patients with different cognition: HC (7.23 ± 2.42 ng/ml), PD-NC (6.83 ± 2.13 ng/ml), and PD-MCI (7.23 ± 2.40 ng/ml) (*F* = 0.337, *p* = 0.714, Figure 2B). Multiple linear regression model showed that baseline CSF sTREM2 was not correlated with MoCA total score in the whole cohort (β = −0.044, *p* = 0.438) or in PD group (β = −0.063, *p* = 0.368) or in HC group (β = −0.042, *p* = 0.708). Even so, we found that elevated CSF sTREM2 were associated with higher levels of CSF α-syn, Aβ_1−42_, P-tau, and T-tau in HC group, PD group, and the whole cohort (all *p* < 0.05, Supplementary Figure S4).

### The Prediction of Longitudinal Changes in Cognitive Decline Using Baseline CSF sTREM2

**Figure 3:**
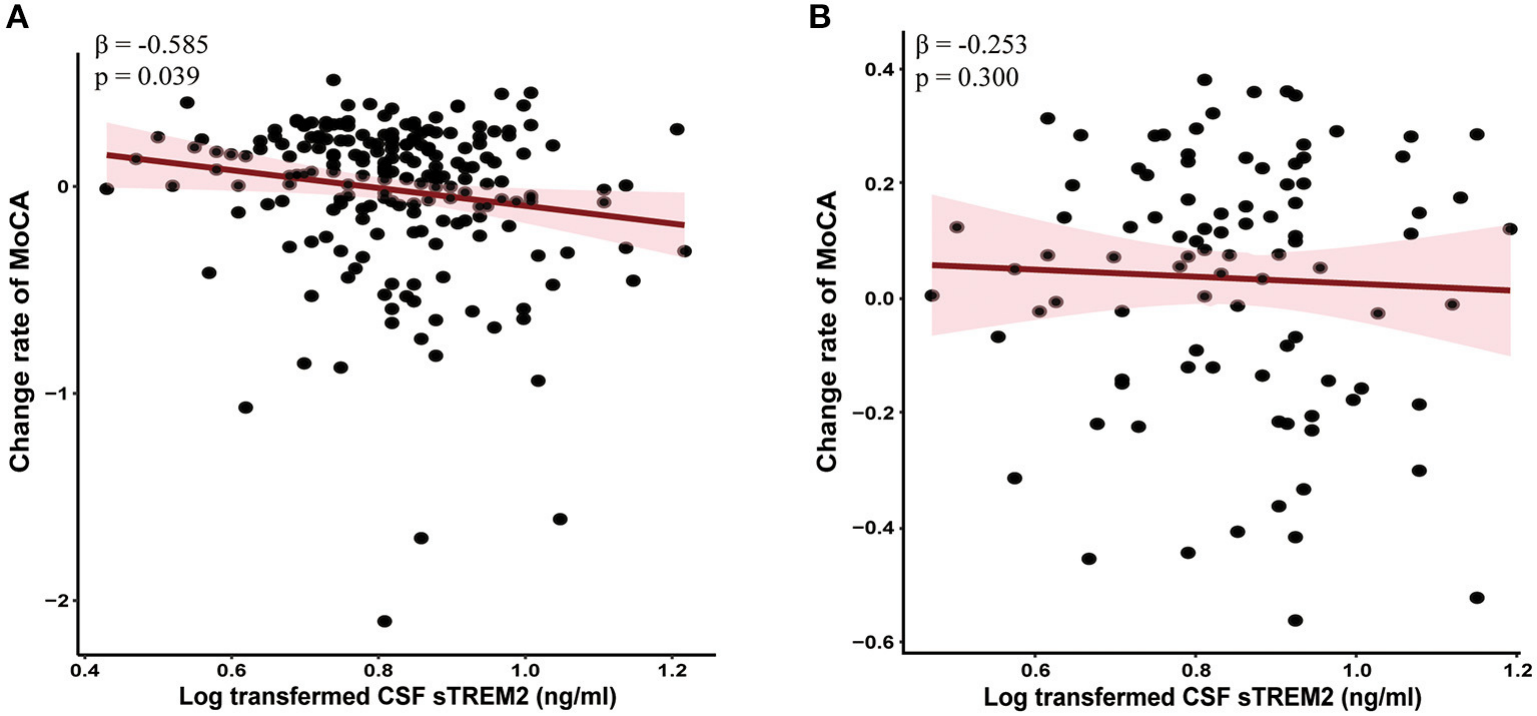
Higher baseline CSF sTREM2 had faster global cognitive declines in PD group **(A)** but not HC group **(B)**. The normalized regression coefficients (β) and *p*-values computed by mixed-effect linear models after adjustment for age, gender, educational level, and *APOE* ε*4* carrier status. CSF, cerebrospinal fluid; sTREM2, soluble triggering receptors expressed on myeloid cells 2; HCs, healthy controls; PD, Parkinson's disease.

Linear mixed-effects models showed that higher baseline CSF sTREM2 were correlated with greater decline in global cognition (MoCA, β = −0.585, *p* = 0.039, Figure 3A), episodic memory (i.e., HVLT, *p* < 0.05), language (SFT, β = −1.745, *p* = 0.044), processing speed/attention (SDMT, β = −1.655, *p* = 0.047), and visuospatial functioning (JoLO, β = −0.391, *p* = 0.013) in PD group. However, no associations were found between baseline CSF sTREM2 with the change in global cognition (MoCA, β = −0.253, *p* = 0.300, Figure 3B) and the other cognitive characteristics (Supplementary Table S2) in HC group.

**Figure 4:**
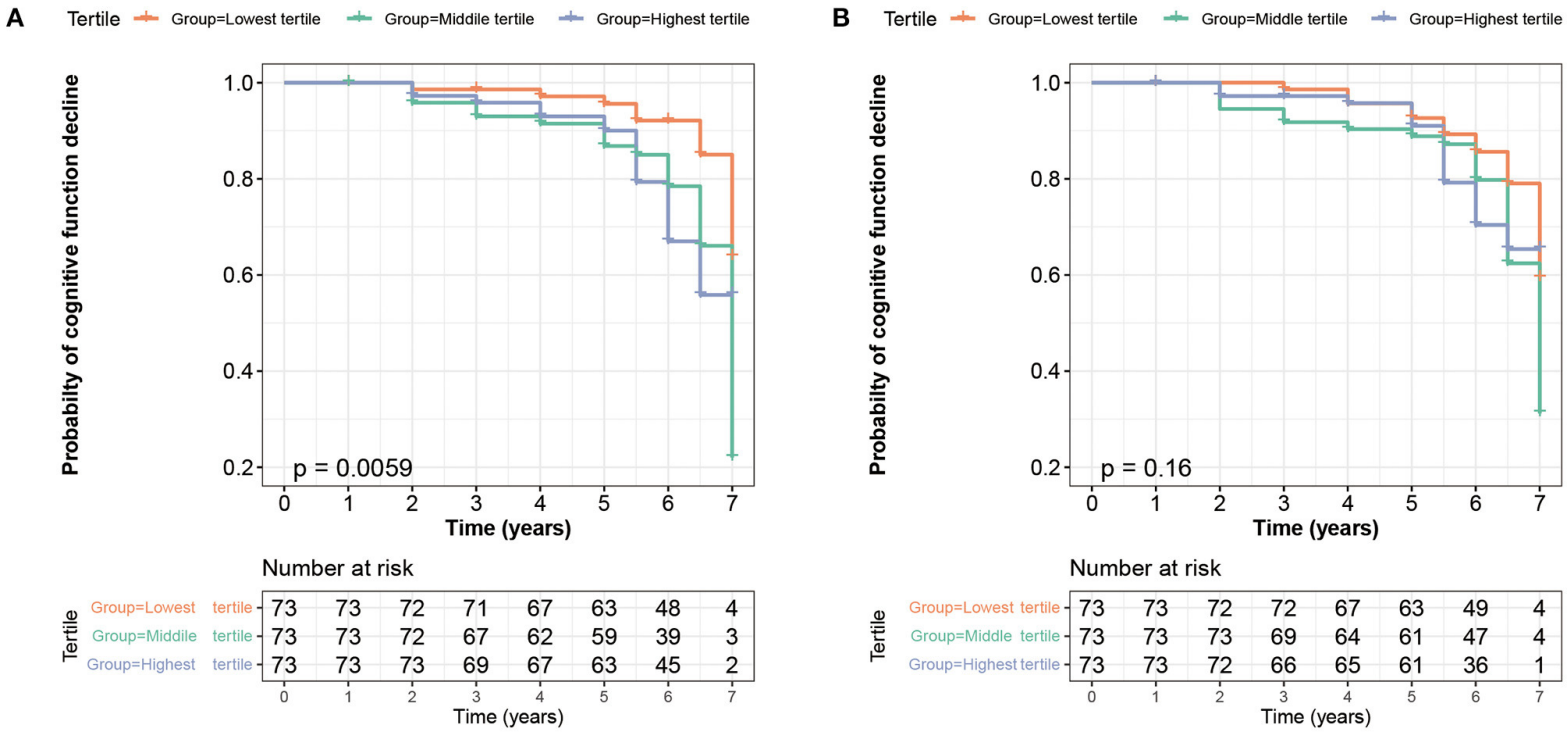
Kaplan–Meier survival curve showed that higher baseline CSF sTREM2 was at a higher risk of cognitive impairment (log-rank *p* = 0.0059, **(A)** whereas the CSF sTREM2 changes did not predict a cognition decline in PD group during 7-year follow-up (log-rank *p* = 0.16, **(B)**. CSF, cerebrospinal fluid; sTREM2, soluble triggering receptors expressed on myeloid cells 2; PD, Parkinson's disease.

With a mean follow-up of 5.51 ± 1.31 years, 23.51% of participants (75/319) suffered from a cognition decline, including 50 patients with PD and 25 healthy controls. To assess the ability of baseline CSF sTREM2 in prediction of conversion risk from normal cognition to MCI or from MCI to dementia condition, participants were divided into three groups based on tertiles of baseline CSF sTREM2. Cox proportional-hazards models showed that patients with the highest tertile (HR = 2.833, 95% CI: 1.226–6.547, *p* = 0.015) and the middle tertile (HR = 2.426, 95% CI: 1.023–5.754, *p* = 0.044) had a higher risk of cognitive conversion than those with lowest tertile in PD group (Table 2). The results of a Kaplan–Meier curves are revealed in Figure 4A. In the whole cohort, patients with the highest tertile had an increased risk of cognitive conversion compared with those in the lowest tertile (HR = 2.466, 95% CI: 1.254–4.852, *p* = 0.009). In HC group, no difference was found in conversion risk of cognition among groups (Table 2). The results of a Kaplan–Meier curves of the whole cohort and HC group are revealed in Supplementary Figures S5A,B.

**Table 2:** Progression risk from normal cognition to MCI or from MCI to dementia.

	**Lowest tertile**	**Middle tertile**		**Highest tertile**	
		**HR (95%CI)**	**p**	**HR (95%CI)**	***p***
**The whole cohort**					
Baseline CSF sTREM2	Reference	1.866 (0.925-3.764)	0.082	2.466 (1.254-4.852)	**0.009**
		Reference		1.298 (0.758- 2.224)	0.342
Change rate of sTREM2	Reference	1.478 (0.805-2.712)	0.208	1.829 (0.990-3.380)	0.054
		Reference		1.197 (0.692- 2.071)	0.521
**PD group**					
Baseline CSF sTREM2	Reference	2.426 (1.023-5.754)	**0.044**	2.833 (1.226-6.547)	**0.015**
		Reference		1.168 (0.608–2.242)	0.641
Change rate of sTREM2	Reference	1.810 (0.860–3.811)	0.118	1.909 (0.888–4.104)	0.098
		Reference		1.215 (0.703–3.698)	0.567
**HC group**					
Baseline CSF sTREM2	Reference	1.555 (0.444–5.442)	0.490	2.302 (0.676–7.836)	0.182
		Reference		1.480 (0.569–3.854)	0.422
Change rate of sTREM2	Reference	1.253 (0.405–3.873)	0.695	1.436 (0.500–4.125)	0.502
		Reference		1.146 (0.407- 3.229)	0.796

*All models were adjusted for age, gender, educational level, and APOE ε4 carrier status*.

*MCI, mild cognitive impairment; CSF, cerebrospinal fluid; sTREM2, soluble triggering receptors expressed on myeloid cells 2; HCs, healthy controls; PD, Parkinson's disease. The bold values represent p < 0.05*.

### The Prediction of Longitudinal Changes in Cognitive Decline Using Longitudinal CSF sTREM2

During a 4-year follow-up period, there was less change in CSF sTREM2 (Supplementary Figure S6). To assess the ability of CSF sTREM2 changes in the prediction for conversion risk from normal cognition to MCI or from MCI to dementia condition, participants were divided into three groups based on tertiles of CSF sTREM2 change rate. In PD group, Kaplan–Meier analysis showed that there was no difference among groups (*p* = 0.16, Figure 4B), whereas Cox proportional-hazards models showed that patients with the highest tertile had a trend to correlate with the risk of cognitive conversion compared to the lowest tertile (HR = 1.909, 95% CI: 0.888–4.104, *p* = 0.098, Table 2). In the whole cohort and HC group, no correlation was found in CSF sTREM2 with the risk of cognitive conversion in Cox proportional-hazards models and Kaplan–Meier analysis (Supplementary Figures S5C,D).

### Difference in CSF sTREM2 Level Between Biomarker Categories

To assess the changes in CSF sTREM2 during the pathology of PD, the participants were divided into groups by A/T classification framework. When the whole cohorts were divided into two groups according to CSF Aβ_1−42_ (A+ and A–) or CSF p-tau (T+ and T–) or CSF α-syn (S+ and S-), we found that A+ group had a lower CSF sTREM2 than A- group, T+ group had a higher CSF sTREM2 than T- group, and S+ group had a lower CSF sTREM2 than S- group (Supplementary Figures S7A–C). These differences remained significant after adjusting for age, gender, education level, and *APOE* ε*4* carrier status.

**Figure 5:**
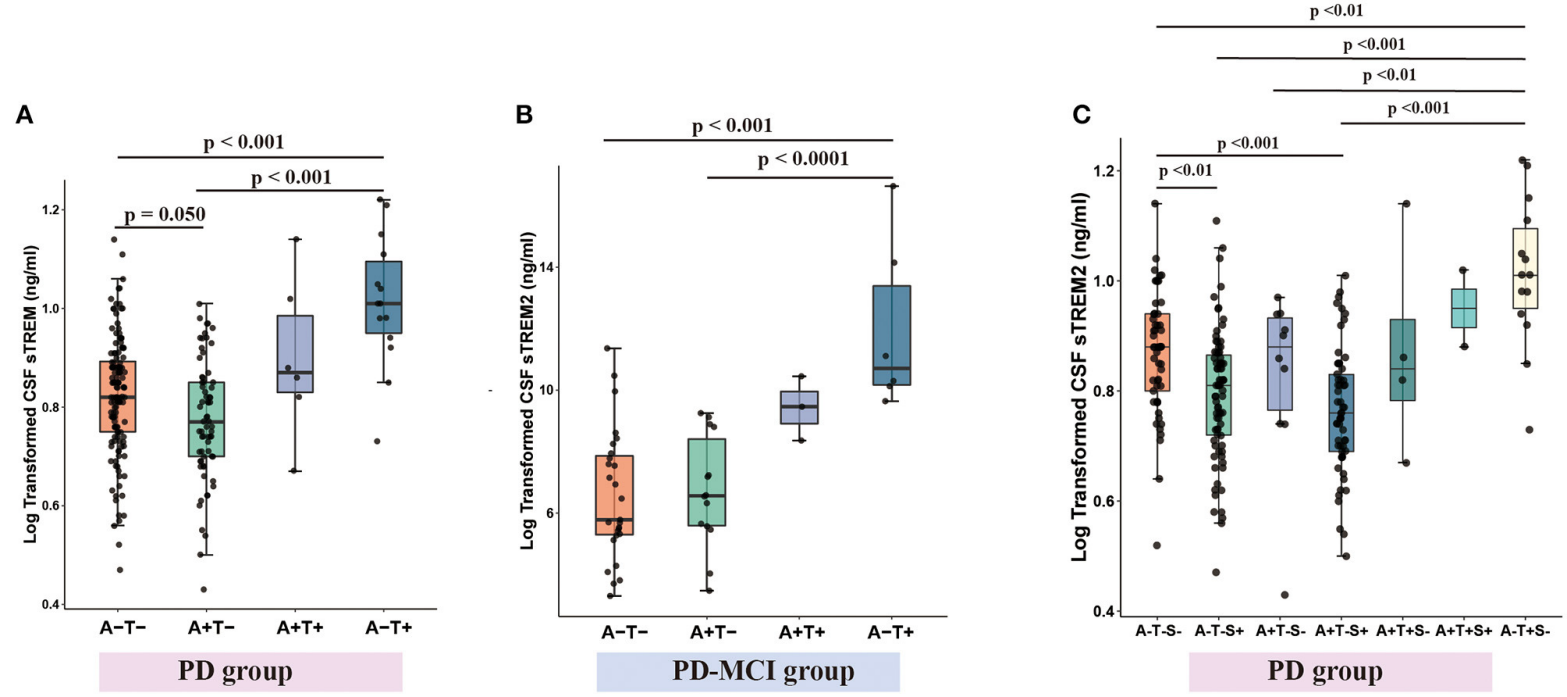
CSF sTREM2 levels within A/T classification framework with or without CSF α-syn (S) in PD group. CSF sTREM2 was lowest in A-T- group and highest in A-T+ group in PD group **(A)**. CSF sTREM2 was highest in tau pathology in PD-MCI group **(B)**. With the addition of CSF α-syn (S) to A/T framework, CSF sTREM2 was lowest in A+T-S+ group, then higher in A-T-S+ group, and highest in A-T+S- group **(C)**. *p*-values were assessed by a one-way ANCOVA, and significant *p*-values after Bonferroni corrected *post-hoc* pairwise comparisons are marked. CSF, cerebrospinal fluid; sTREM2, soluble triggering receptors expressed on myeloid cells 2; PD, Parkinson's disease; A+, Aβ pathology (defined as low CSF Aβ1–42); T+, tau pathology (defined as high CSF P-tau); S+, α-syn pathology (defined as low CSF α-syn); PD, Parkinson's disease; MCI, mild cognitive impairment.

When PD group, the whole cohort and HC group were divided into four groups according to CSF Aβ_1−42_ (A+ or A–) and CSF p-tau (T+ or T–), the concentration of CSF sTREM2 was lowest in A+T- group, higher in A+T+ group and highest in A-T+ group (Figure 5A and Supplementary Figures S7D,E). Taking into account the clinical stages, the same pattern was found in PD-NC group (Supplementary Figure S7F). Although higher CSF sTREM2 were found in A-T+ group in PD-MCI group, it was not lower in A+T- group (Figure 5B).

When with the addition of α-syn to A/T classification framework, PD group and the whole cohort and HC group were divided into eight groups according to CSF Aβ_1−42_ (A+ or A–) and CSF p-tau (T+ or T–) and α-syn (S+ and S–), and the concentration of CSF sTREM2 was lowest in A+T-S+ group, then higher in A-T-S+ group, and highest in A-T+S- group (Figure 5C and Supplementary Figures S7G,H). Taking into account the clinical stages, the similar pattern was found in PD-NC group (Supplementary Figure S7I). However, the number of patients (*n* = 48) in the PD-MCI group was too small to be divided into eight groups for statistical analysis.

## Discussion

In this study, we comprehensively analyzed the expression of CSF sTREM2 in patients with PD and its association with cognitive decline. The primary results showed that patients with PD with higher baseline CSF sTREM2 had faster cognitive decline at follow-ups compared to healthy controls. Moreover, the use of A/T classification framework in combination with α-syn showed that decreased CSF sTREM2 were associated with Aβ pathology and α-syn pathology, whereas elevated CSF sTREM2 were associated with tau pathology. However, the concentration of CSF sTREM2 did not differ between the patients with PD and the healthy controls or between PD clinical subgroups (PD-NC and PD-MCI). These findings suggested that CSF sTREM2 change dynamically with alterations in CSF biomarkers of brain amyloidosis and neuronal injury, and CSF sTREM2 could be a predictive indicator for cognitive decline rather than a specific diagnostic biomarker for patients with PD.

Triggering receptor expressed on myeloid cells two is a surface receptor that expressed on microglia and is involved in promoting phagocytosis of apoptotic neurons and misfolded proteins and inhibiting neuroinflammation (Deczkowska et al., 2020). In PD animal models, TREM2 deficiency aggravates α-syn-induced neuroinflammation and neurodegeneration, and its overexpression remarkably attenuates neuroinflammation and protects DA neurons, indicating TREM2 may contribute to microglial recognition and endocytosis of α-syn (Ren et al., 2018; Zhang et al., 2018; Guo et al., 2019). sTREM2 in CSF is a surrogate measure of TREM2-triggered microglial activity, and recent studies noted that it increased in early symptomatic stages of patients with AD and aging adults (Henjum et al., 2016; Suárez-Calvet et al., 2016). Notably, cross-sectional studies showed that CSF sTREM2 was elevated in PD and correlated with CSF α-syn (Peng et al., 2020; Mo et al., 2021). However, although baseline CSF sTREM2 was associated with CSF α-syn and age in this study, it was not different between healthy controls and patients with PD or PD clinical subgroups (PD-NC and PD-MCI), which was consistent with the other studies from PPMI (Wilson et al., 2020; Bartl et al., 2021). Our results differed from these cross-sectional studies, possibly because that patients with PD in our study were at the early stage of new diagnosis, which may initially involve a smaller number of neuron damage than AD, so that synaptic damage and neuroinflammation may not be widespread enough to be reflected in CSF (Bartl et al., 2021). Moreover, a two-sample Mendelian randomization study showed that genetically determined CSF sTREM2 was not associated with PD (Dong et al., 2022). Additionally, CSF sTREM2 was elevated in PD subgroups with a positive tau CSF biomarker (Wilson et al., 2020), suggesting that CSF sTREM2 may be pathologically specific and dynamically variable. Therefore, whether CSF sTREM2 could be a diagnostic biomarker for PD needs further confirmation.

Cognitive dysfunction is a common feature of PD with an overall incidence of about 30% (Emre et al., 2007). It is estimated that the incidence of dementia in PD is increased up to six times than that in the general population (Emre et al., 2007). The presence of cognitive decline or dementia in patients with PD not only exacerbates the disabilities and independence, but also reduces quality of life and survival time (Rosenthal et al., 2010). Thus, identifying biomarkers to predict cognitive progression in PD is crucial for its clinical intervention. Recently, studies from Alzheimer's Disease Neuroimaging Initiative (ADNI) reported that higher baseline CSF sTREM2 predicted a reduced rate of cognitive decline and clinical decline in patients with AD (Ewers et al., 2019; Franzmeier et al., 2020). However, in this study, a higher baseline CSF sTREM2 was associated with a greater cognitive decline in patients with PD (Bartl et al., 2021). Our results that are contrary to the previous studies may be related to several potential pathways. On the one hand, sTREM2 could competitively bind TREM2 ligand as a decoy receptor and inhibit the neuroprotective function of TREM2 receptor (Konishi and Kiyama, 2018). On the other hand, sTREM2 promoted proinflammatory responses of microglia *via* PI3K-NF-κB pathway and triggers inflammatory cytokine release, which may cause neuronal death (Zhong et al., 2017; Hickman et al., 2018). Additionally, inhibiting shedding and releasing of TREM2 and maintaining protein levels in TREM2 improved cognitive function in APP/PS1 mice, suggesting that TREM2, but not sTREM2, has a protective effect against inflammation and neuronal damage (Zhang et al., 2022). These findings further support that CSF sTREM2 may be a potential biomarker of TREM2-mediated microglia function and neuronal injury.

Pathological studies show that PD and AD share a certain degree of neuropathological overlap (Irwin et al., 2013). Up to 50% PD dementia has sufficient Aβ plaques and tau tangles, and Aβ plaques promote seeding and spreading of α-syn and Tau (Irwin et al., 2013; Bassil et al., 2020). Thus, protein co-aggregation and mutual triggering leading to relevant neurons loss is associated with cognitive decline and motor disorder (Tsuang et al., 2013; Heywood et al., 2015). Recently, CSF sTREM2 was found to be associated with CSF α-syn and tau-pathology in patients with PD (Wilson et al., 2020; Mo et al., 2021). Similar to these studies, this study showed that the CSF sTREM2 was positively associated with CSF α-syn, T-tau, and P-tau in patients with PD and healthy controls, suggesting that CSF sTREM2 may indeed reflect neuronal injury. However, a positive correlation between CSF sTREM2 and Aβ_1−42_ in our study is confusing. Although this result is supported by two recent studies (Bekris et al., 2018; Ma et al., 2020), most studies describe no association between CSF sTREM2 and CSF Aβ_1−42_ (Suárez-Calvet et al., 2019; Wilson et al., 2020). The reasons for the differences are unknown. It has been described that increased Aβ_1−42_ may reflect an imbalance between Aβ production and clearance, rather than selective changes in Aβ42 associated with Aβ deposition (Mawuenyega et al., 2010). Moreover, amyloid formation depends on seeding: the local concentration of Aβ monomers must reach a critical threshold before fibril formation to begin (Jarrett and Lansbury, 1993). In this study, the included patients with PD were drug-naïve early-stage and had no symptom of dementia. In addition, CSF Aβ_1−42_, T-tau, and P-tau are lower in patients with PD than those in HC (Kang et al., 2016). Therefore, positive correlation between CSF sTREM2 and Aβ_1−42_ at baseline may reflect a very early pre-symptomatic stage of dementia. Our findings are preliminary and require further examine in other cohorts and in patients with PD with dementia.

Consistent with previous studies showing the dynamic changes in CSF sTREM2 in AD, we found that elevated CSF sTREM2 was associated with tau pathology even in the absence of Aβ pathology, whereas decreased CSF sTREM2 was associated with Aβ pathology in patients with PD (Suárez-Calvet et al., 2019; Ma et al., 2020). Moreover, in addition to α-syn to A/T classification framework, the reduction in CSF sTREM2 seems to be more significant in the presence of both Aβ and α-syn pathologies, whereas elevated CSF sTREM2 was still associated with tau pathology. These results demonstrated that CSF sTREM2 has a complex expression pattern in PD. Several previous studies have consistently demonstrated that the increased CSF sTREM2 is caused by microglial activation in response to tau pathology (Lue et al., 2015; Suárez-Calvet et al., 2016). In addition, the reduction in CSF sTREM2 in Aβ pathology may be due to microglia initially form a barrier around Aβ plaques and CSF sTREM2 released by microglia is retained within the plaque barrier (Condello et al., 2015; Wang et al., 2016). Another explanation is that although sTREM2 may bind to Aβ and exert effects, CSF sTREM2 may not reflect such activation in the absence of downstream induced tauopathy (Zhao et al., 2018; Fan et al., 2019). However, the mechanisms underlying that CSF sTREM2 was decreased in α-syn pathology (defined as low CSF α-syn) are unclear. α-syn in PD has similar mechanisms of aggregation and degradation as Aβ in AD (Cong et al., 2021). They share a GAV motif in peptides that may be associated with promoter protein aggregation and similar degradation mechanisms including intracellular and extracellular aggregation, microglial activation, and endocytosis (Lin et al., 2006; Schechter et al., 2020). Therefore, we speculate that TREM2 may be involved in microglial recognition and endocytosis of α-syn. However, we are cognizant of the fact that this study is an observational study that makes it difficult to study specific mechanism about the association between CSF sTREM2 and α-syn. Additionally, future studies are needed to validate these findings. Nevertheless, elevated CSF sTREM2 in tau pathology suggests that it could use to monitor cognitive progression in PD and may be a promising outcome measurement for future clinical trials of cognitive decline and neuroinflammation.

The strength of this study was to demonstrate the association of CSF sTREM2 with cognitive decline and uncover its essential roles for monitoring neuronal injury in an international, multicenter prospective clinical cohort. However, there were some limitations. First, the relatively small sample size may affect the statistical power of differences between groups, and thus, a larger sample size study is needed to verify our findings. Second, individual with potential TREM2 mutations was not tested in this study, which are known to affect the expression of CSF sTREM2. However, the prevalence of TREM2 mutations was so rare in patients with PD that unlikely to affect our results. Finally, A/T/ (N) classification system with the addition of α-syn was used for the first time to detect the dynamic changes in CSF sTREM2 in PD, but the cutoff values of CSF Aβ1–42, P-tau, T-tau, and α-syn have not been established in PD. Therefore, whether A/T/ (N) biomarker classification system is suitable for the patients with PD needs to be further studied, especially after the addition of α-syn.

## Conclusion

In conclusion, our findings demonstrate that CSF sTREM2 is a promising predictive biomarker to monitor cognitive progression rather than a reliable diagnostic biomarker for Parkinson's disease. Furthermore, the levels of CSF sTREM2 changed dynamically with α-syn and Aβ pathology and tau pathology, suggesting a meaningful measurement for the TREM2-triggered microglial activity and neuronal injury in future clinical trials. Further prospective studies with larger sample sizes are urgently needed to confirm our findings.

## Data Availability Statement

The original contributions presented in the study are included in the article/Supplementary Material, further inquiries can be directed to the corresponding author.

## Ethics Statement

The studies involving human participants were reviewed and approved by Parkinson's Progression Markers Initiative. The patients/participants provided their written informed consent to participate in this study.

## Author Contributions

QQ and ZX conceived, designed the research, had primary responsibility for writing, editing, and revising the manuscript. HW, DW, and JinL helped to analyze the data. YQ, JZ, and JiaL helped to revise the manuscript. ZX provided financial support for this work. All authors read and approved the final manuscript.

## Funding

This work was funded by the National Natural Science Foundation of China (Grant Nos. 81771376 and 91849121).

## Conflict of Interest

The authors declare that the research was conducted in the absence of any commercial or financial relationships that could be construed as a potential conflict of interest.

## Publisher's Note

All claims expressed in this article are solely those of the authors and do not necessarily represent those of their affiliated organizations, or those of the publisher, the editors and the reviewers. Any product that may be evaluated in this article, or claim that may be made by its manufacturer, is not guaranteed or endorsed by the publisher.
