# Supplementary material for: The Association of CSF sTREM2 With Cognitive Decline and Its Dynamic Change in Parkinson's Disease: Analysis of the PPMI Cohort
Source: Front Aging Neurosci. 2022 Jun 16;14:892493. doi: 10.3389/fnagi.2022.892493 (PMC9245456; doi:10.3389/fnagi.2022.892493)
Supplement: Supplementary file 1 [file Data_Sheet_1.docx]

**SUPPLEMENTARY INFORMATION**

**SUPPLEMENTARY FIGURE LEGENDS**


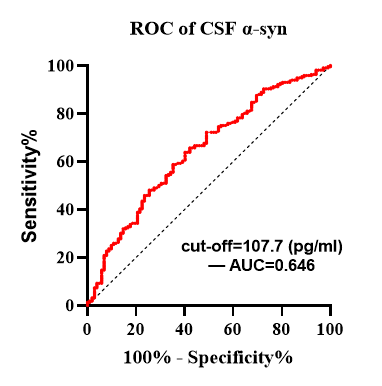


**Supplementary Figure 1.** ROC analyses showed that optimal diagnostic cut-off values for CSF α-syn was 107.7 pg/ml in discriminating PD patients from healthy controls. The area under the curve (AUC) was 0.646 (95% CI: 0.5818-0.7110).


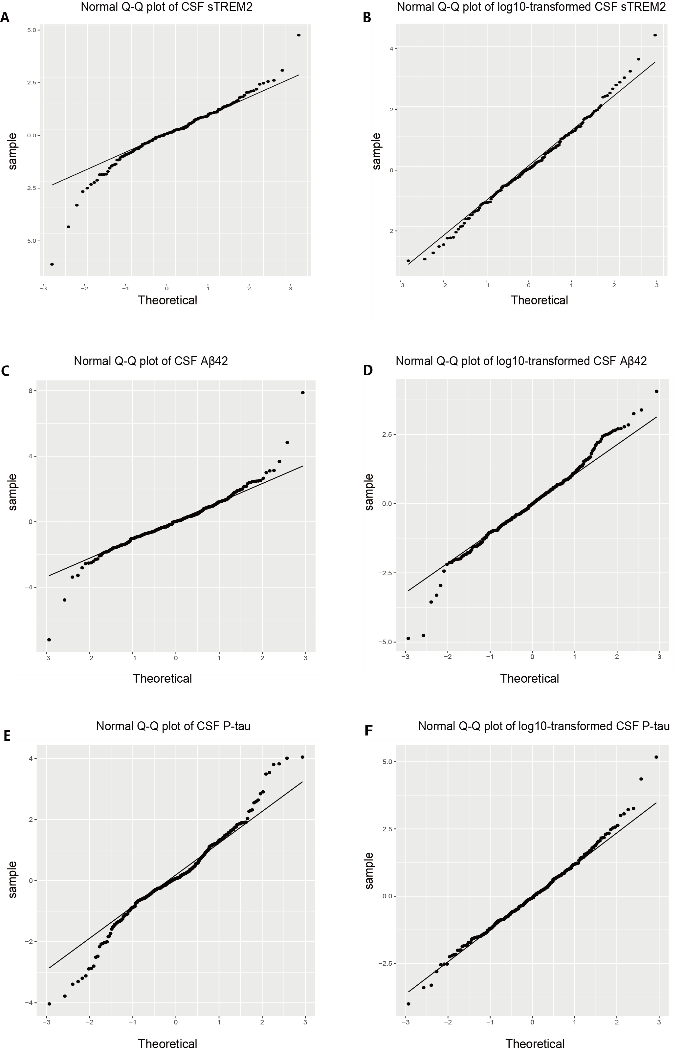
 **
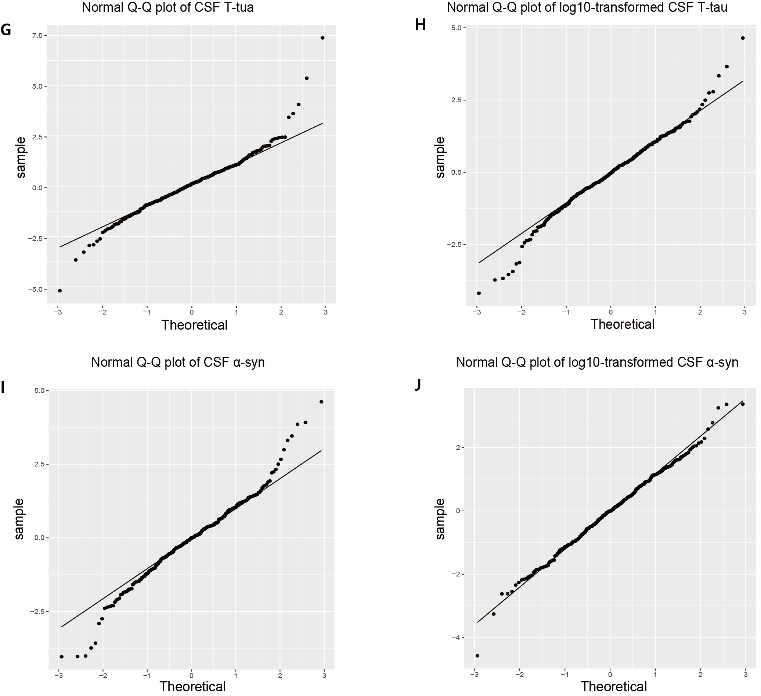
**

**Supplementary Figure 2.** Sample quantiles were plotted following those theoretical quantiles under the null hypothesis (x-axis). The Quantile-Quantile plot shows that CSF sTREM2 (A-B), CSF Aβ42 (C-D), CSF p-tau (E-F), CSF t-tau (G-H), and CSF α-syn (I-J) data did not conform to the normal distribution and log10-transformed these data conforms to the normal distribution.


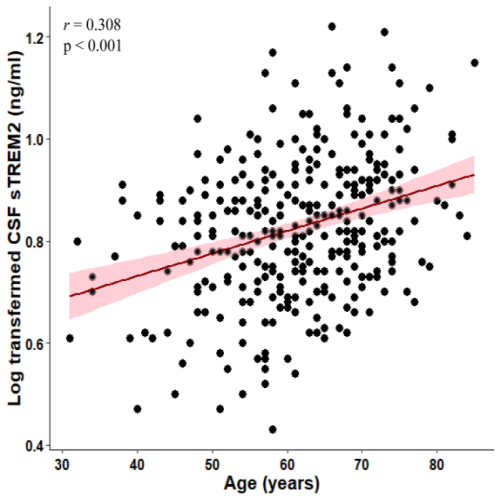


**Supplementary Figure 3.** CSF sTREM2 was associated with age in the whole cohort (r=0.308, p <0.001). The correlation coefficient (r) and p-values computed by Spearman rank correlation.


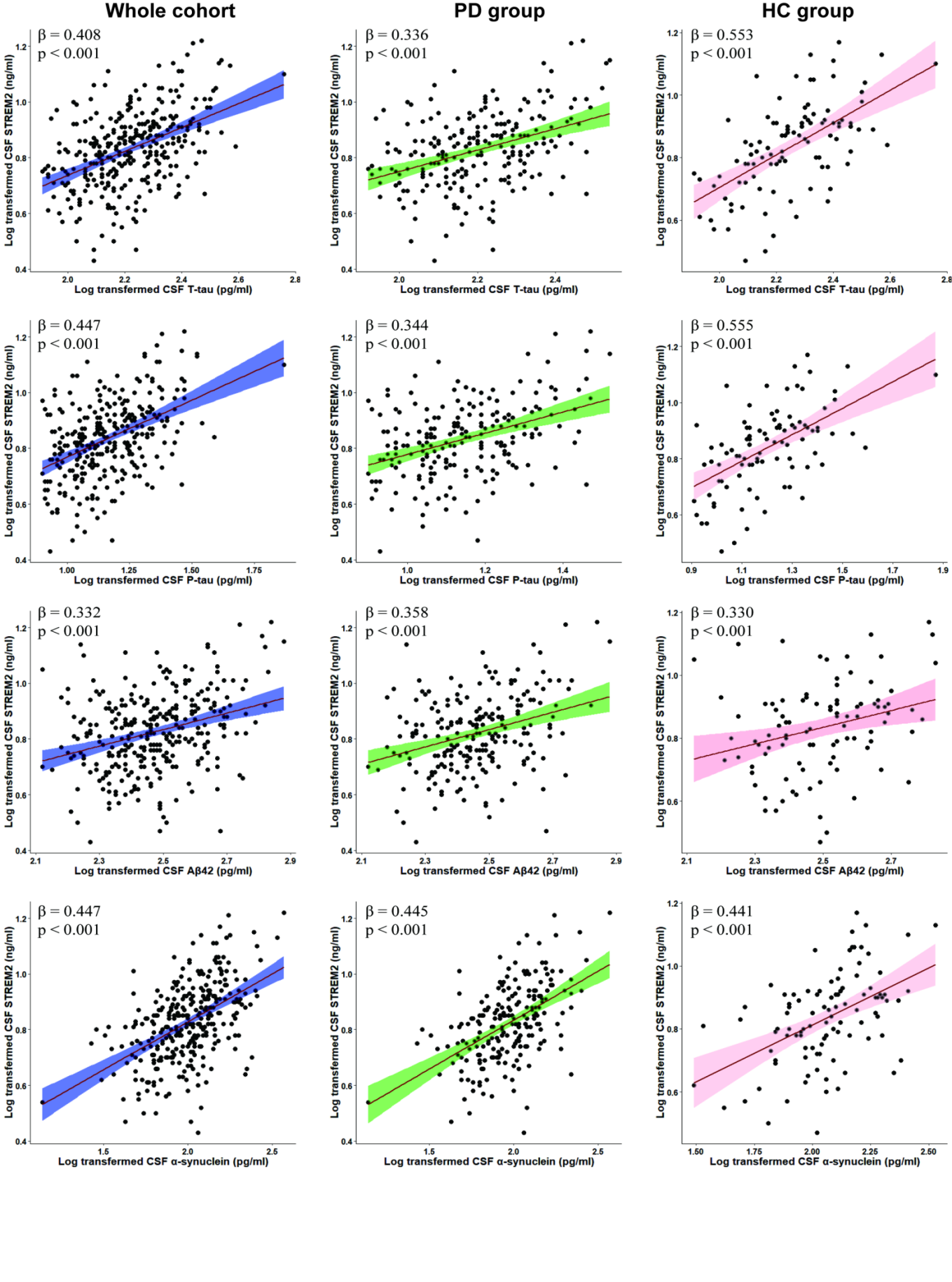


**Supplementary Figure 4.** Baseline associations of CSF sTREM2 with Aβ_1–42_, T-tau, P-tau, and α-syn in the whole cohort, PD group, and HC group. The normalized regression coefficients (β) and p-values computed by multiple linear regression after adjustment for age, gender, educational level, and *APOE ε4* carrier status.

**
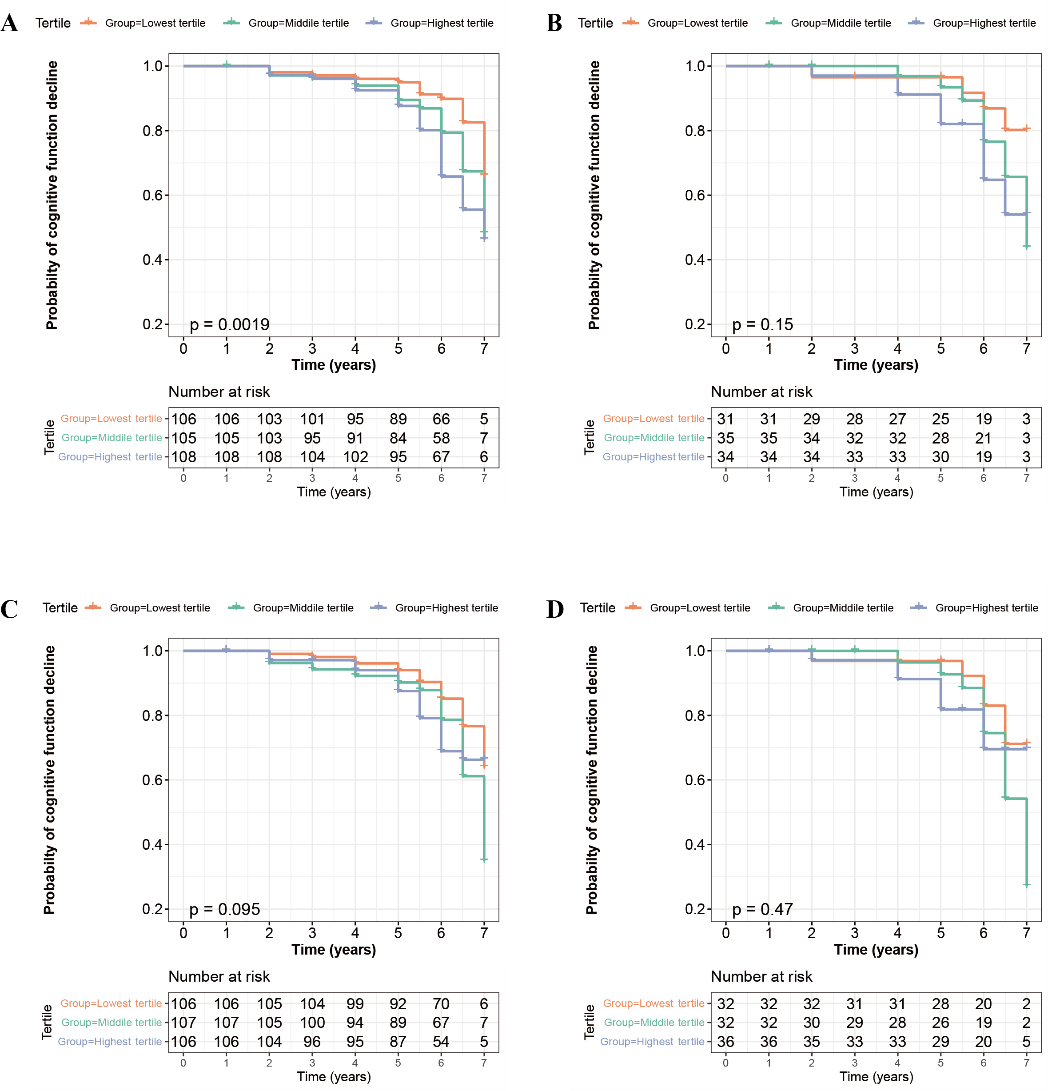
**

**Supplementary Figure 5.** Kaplan-Meier survival curve for conversion from normal cognition converted to MCI or from MCI to dementia during 7-years follow-up. In whole cohort, higher baseline CSF sTREM2 were at a higher risk of cognitive impairment progression (Log-rank p=0.0019; A), while the CSF sTREM2 changes did not predict a cognition decline (p=0.15; B). In HC group, baseline CSF sTREM2 (Log-rank p=0.095; C) and CSF sTREM2 changes (Log-rank p=0.47; D) were not predictive of cognitive decline.


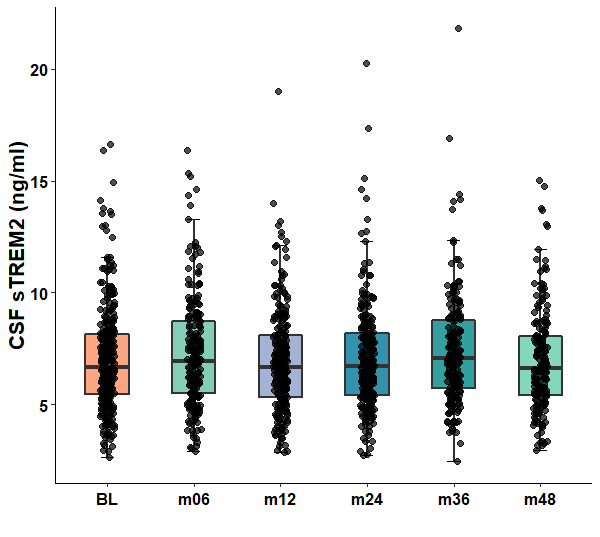


**Supplementary Figure 6.** The change of CSF sTREM2 levels during follow-up**.**


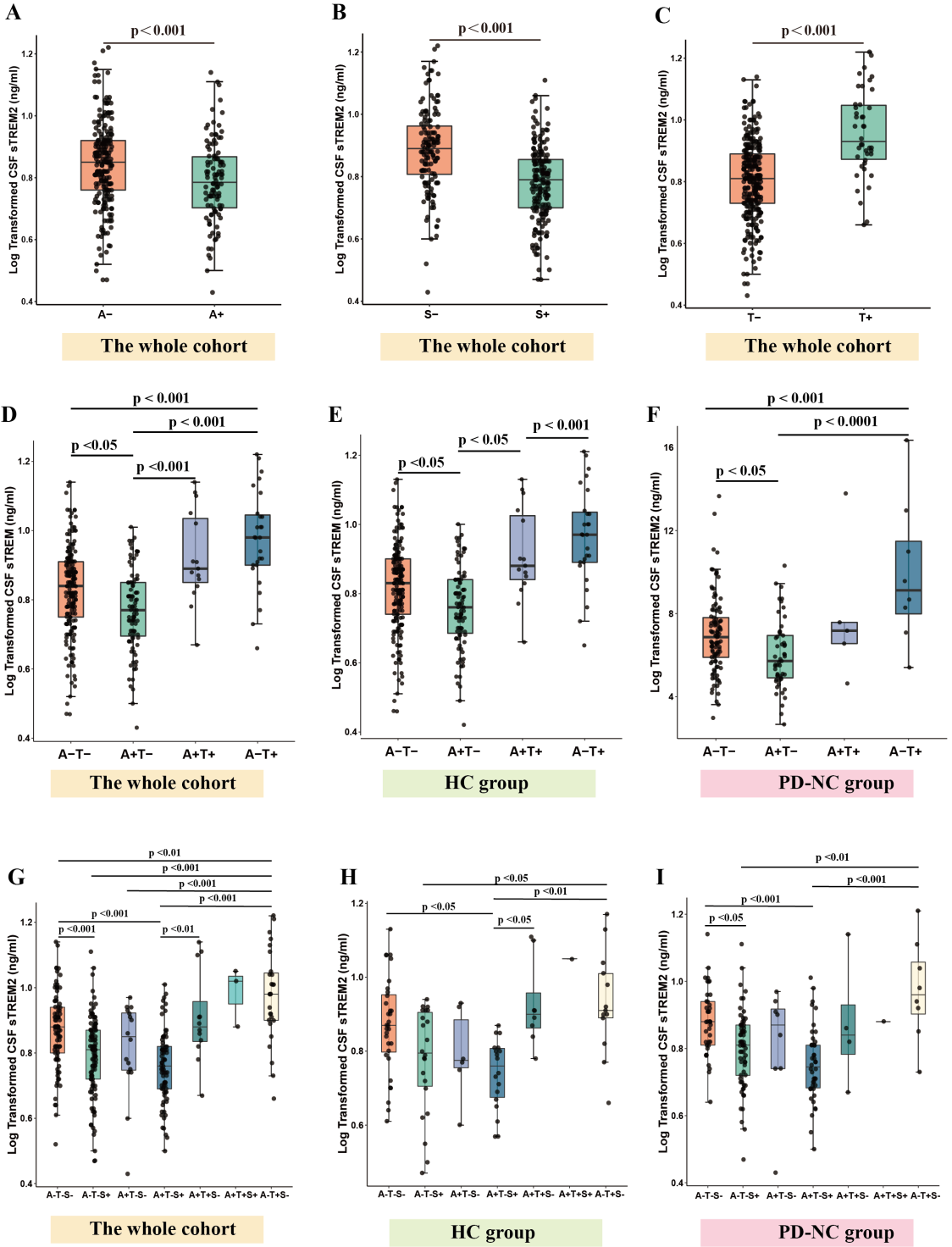


**Supplementary Figure 7.** **Difference in CSF sTREM2 level between biomarker categories**.

CSF sTREM2 were decreased in A+ group and S+ group, while increased in T+ group in the whole cohort (A-C). CSF sTREM2 within A/T classification framework in the whole cohort, HC group and PD-NC group. The same pattern was found in three group that CSF sTREM2 was lower in A+T+ group and was higher in A+T group or A-T+ group (E-F). CSF sTREM2 within A/T classification framework with addition of CSF α-syn in the whole cohort, HC group and PD-NC group. Similar pattern was found in three groups that CSF sTREM2 was lowest in A+T-S+ group, and highest in A-T+S- group (H-I). p-values were assessed by a one-way ANCOVA, and significant p-values after Bonferroni corrected post hoc pairwise comparisons are marked.

Abbreviations: A+, Aβ pathology (defined as low CSF Aβ_1–42_); T+, tau pathology (defined as high CSF P-tau); S+, α-syn pathology (defined as low CSF α-syn); PD, Parkinson’s disease; HC, healthy controls.

**Supplementary Table 1. Follow-up data of longitudinal CSF biomarkers and cognitive assessments.**

| **Timepoints (months)** | **BL** | **M6** | **M12** | **M24** | **M36** | **M48** | **M60** | **M72** | **M84** |
| --- | --- | --- | --- | --- | --- | --- | --- | --- | --- |
| sTREM2 | 319 | 212 | 254 | 245 | 190 | 188 |  |  |  |
| Aβ_1-42_ | 319 | 243 | 284 | 289 | 227 |  |  |  |  |
| T-tau | 319 | 243 | 284 | 289 | 227 |  |  |  |  |
| P-tau | 319 | 243 | 284 | 289 | 227 |  |  |  |  |
| α-syn | 319 | 212 | 254 | 245 | 190 | 188 |  |  |  |
| MoCA | 319 | 299 | 300 | 290 | 274 | 251 | 170 | 193 | 106 |
| JoLO | 319 | 299 | 300 | 291 | 275 | 251 | 167 | 190 | 103 |
| LNS | 319 | 299 | 300 | 291 | 275 | 251 | 167 | 190 | 103 |
| SDMT | 319 | 299 | 300 | 291 | 275 | 251 | 167 | 190 | 103 |
| Semantic Fluency Test | 319 | 299 | 300 | 291 | 275 | 251 | 167 | 190 | 103 |
| HVLT Total Recall | 319 | 299 | 300 | 291 | 275 | 251 | 167 | 190 | 103 |
| HVLT Delayed Recall | 319 | 299 | 300 | 291 | 275 | 251 | 167 | 190 | 103 |
| HVLT Retention | 319 | 299 | 300 | 291 | 275 | 251 | 167 | 190 | 103 |
| HVLT RD | 319 | 299 | 300 | 291 | 275 | 251 | 167 | 190 | 103 |

The data are number of different measures at each study visit.

Abbreviations: sTREM2, soluble triggering receptors expressed on myeloid cells 2; Aβ_1-42_, Amyloid_1-42_; T-tau, Total tau; P-tau, Phosphorylated tau; α-syn, α-synuclein; MoCA, Montreal Cognitive Assessment; JoLO, Benton Judgment of Line Orientation Score; LNS, Letter Number Sequencing; SDMT, Symbol Digit Modality Test; HVLT, Hopkins Verbal Learning Test; HVLT RD, HVLT Recognition Discrimination.

**Supplementary Table 2. The association of baseline and** **longitudinal change of CSF sTREM2 with cognition assessments and CSF biomarkers.**

|  | **Baseline CSF sTREM2** | | | | |  | **Change rate of CSF sTREM2** | | | | |
| --- | --- | --- | --- | --- | --- | --- | --- | --- | --- | --- | --- |
|  | **HC group** | |  | **PD group** | |  | **HC group** | |  | **PD group** | |
|  | **β** | **p** |  | **β** | **p** |  | **β** | **p** |  | **β** | **p** |
| Aβ_1-42_ | -49.256 | **0.013** |  | -3.361 | 0.784 |  | 0.228 | **0.023** |  | 0.272 | **<0.001** |
| T-tau | -14.993 | 0.148 |  | 12.364 | **0.063** |  | 0.492 | **<0.001** |  | 0.416 | **<0.001** |
| P-tau | -0.714 | 0.491 |  | 0.844 | 0.149 |  | -0.445 | **<0.001** |  | -0.413 | **<0.001** |
| α-syn | -6.788 | 0.519 |  | 28.223 | 0.205 |  | 0.303 | **0.002** |  | 0.199 | **0.003** |
| MoCA | -0.253 | 0.300 |  | -0.585 | **0.039** |  | 0.035 | 0.729 |  | -0.034 | 0.617 |
| JoLO | -0.065 | 0.756 |  | 0.391 | **0.013** |  | 0.167 | 0.107 |  | 0.063 | 0.368 |
| LNS | 0.098 | 0.679 |  | -0.214 | 0.293 |  | 0.105 | 0.315 |  | -0.063 | 0.374 |
| SDMT | 0.939 | 0.253 |  | -1.655 | **0.047** |  | 0.059 | 0.574 |  | -0.102 | 0.146 |
| Semantic Fluency Test | -1.771 | 0.080 |  | -1.745 | **0.044** |  | -0.165 | 0.111 |  | -0.041 | 0.565 |
| HVLT Total Recall | -0.172 | 0.871 |  | -1.900 | **0.017** |  | 0.001 | 0.990 |  | -0.114 | 0.104 |
| HVLT Delayed Recall | -1.538 | 0.217 |  | -1.973 | **0.030** |  | -0.093 | 0.373 |  | -0.098 | 0.116 |
| HVLT Retention | -1.519 | 0.307 |  | -1.861 | **0.050** |  | -0.139 | 0.181 |  | -0.036 | 0.608 |
| HVLT RD | -1.195 | 0.366 |  | -0.897 | 0.369 |  | -0.117 | 0.263 |  | 0.037 | 0.603 |

The normalized regression coefficients (β) and adjusted P-values were computed by Linear mixed-effects models and multiple linear regression

Adjusted for age, gender, educational level, and *APOE ε4* carrier status.

Abbreviations: sTREM2, soluble triggering receptors expressed on myeloid cells 2; Aβ1-42, Amyloid1-42; T-tau, Total tau; P-tau, Phosphorylated tau; α-syn, α-synuclein; MoCA, Montreal Cognitive Assessment; JoLO, Benton Judgment of Line Orientation Score; LNS, Letter Number Sequencing; SDMT, Symbol Digit Modality Test; HVLT, Hopkins Verbal Learning Test; HVLT RD, Recognition Discrimination; UPDRS-III, Unified Parkinson’s Disease Rating Scale part III; H&Y, Hoehn and Yahr.
